# Supplementary material for: Fibronectin-guided migration of carcinoma collectives
Source: Nat Commun. 2017 Jan 19;8:14105. doi: 10.1038/ncomms14105 (PMC5253696; doi:10.1038/ncomms14105)
Supplement: Supplementary Information — Supplementary Figures, Supplementary Table and Supplementary References [file ncomms14105-s1.pdf]

Supplementary Figure 1

a **Multivariate Cox regression analysis of predicting factors for disease-free and overall survival in 435 HNSCC patients**

Factor	Hazard Ratio (95% CI)	*P*-value
**Disease-free survival**		
Clinical stage (Stage III+IV *versus* I+II)	1.721 (1.253 – 2.068)	0.014
pT status (T3+T4 *versus* T1+T2)	1.614 (1.068 – 3.687)	0.021
pN status (N1+N2+N3 *versus* N0)	3.439 (1.325 – 2.592)	0.008
Tumor grade (moderate+poor *versus* well)	1.611 (0.908 – 3.871)	0.059
Fibronectin expression (high *versus* low)	6.811 (1.101 – 21.005)	<0.001
**Overall survival**		
Clinical stage (Stage III+IV *versus* I+II)	1.665 (1.282 – 2.257)	0.026
pT status (T3+T4 *versus* T1+T2)	1.411 (1.012 – 1.898)	0.032
pN status (N1+N2+N3 *versus* N0)	1.724 (1.215 – 1.895)	0.019
Tumor grade (moderate+poor *versus* well)	1.073 (0.529 – 1.213)	0.094
Fibronectin expression (high *versus* low)	2.879 (1.765 – 5.867)	0.001

b

FN staining in whole sections of HNSCC

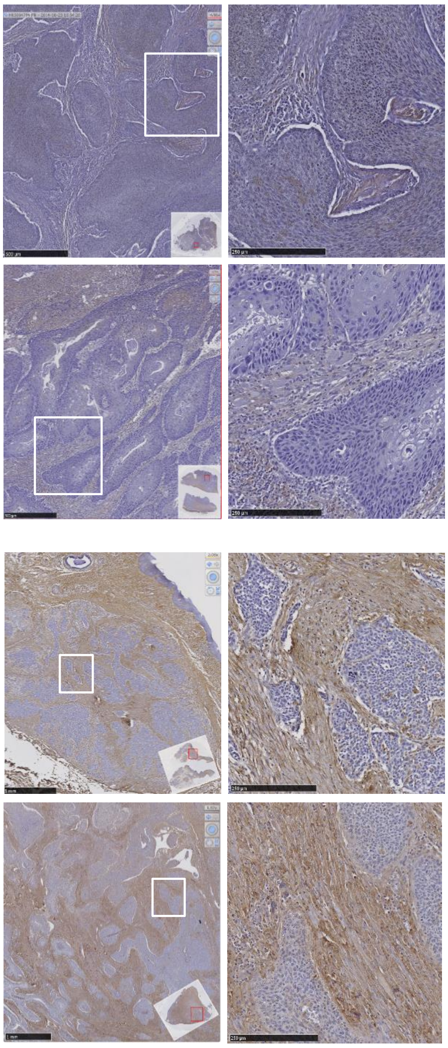

c

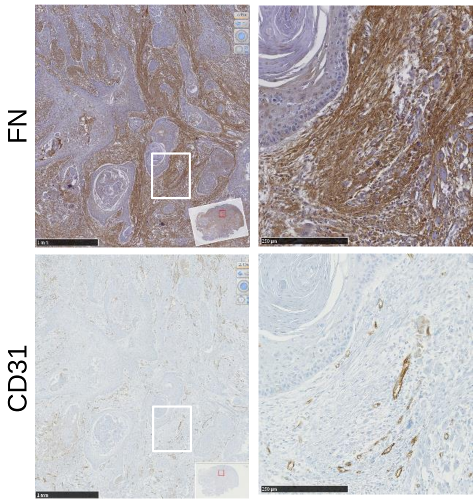

FN expression and predictive value in human HNSCC (a) Multivariate Cox regression analysis of predicting factors for disease-free and overall survival in 435 HNSCC patients. **(b)** Representative immunohistochemical staining of intratumoural FN in whole sections from 4 tumours with low (top) and high (bottom) expression levels. Images on the right (inserts from left images) correspond to the size of TMA histospots (600µm). Scale bar = 250µm for right images and 500µm (top) or 1mm (bottom) for left images. **(c)** Representative staining of FN and CD31 in nearby whole sections from the same tumour. Enlarged insert field is shown on the right (scale bar = 250µm). Note that FN staining in the stroma is not restricted to perivascular regions.

Supplementary Figure 2

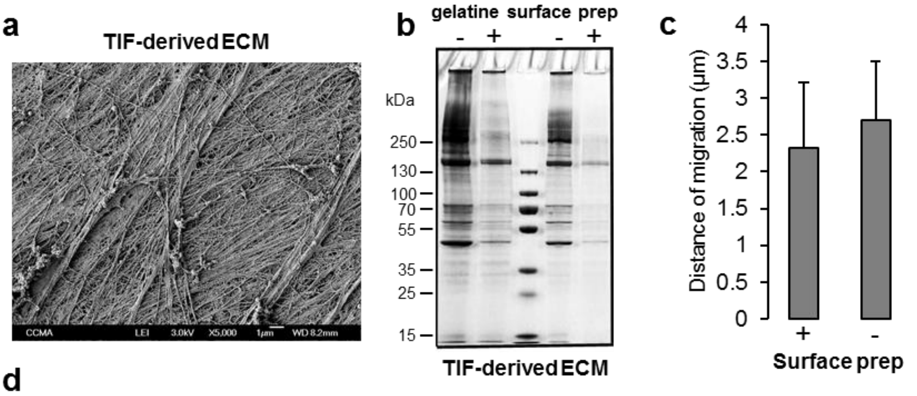

d

Collagens

GeneName	TIF	CAF1	CAF2
COL1A1	7.93	42.42	4.63
COL1A2	3.37	7.39	1.37
COL6A3	1.75	0.78	1.02
COL6A1	1.22	0.68	0.87
COL12A1	0.63	0.35	0.27
COL6A2	0.19	0.3	0.39
COL3A1	0.25	0.2	0.23
COL5A2	0.12	0.14	0.06
COL5A1	0.09	0.1	0.04
COL4A2	0.06		
COL11A1	0.05		
COL2A1	0.04	0.02	
COL4A1	0.03		
COL16A1	0.03	0.01	0.01
COL14A1		0.02	
COL15A1	0.01		
COL18A1	0.01		
COL7A1	0.01		

Proteoglycans

GeneName	TIF	CAF1	CAF2
BGN	0.31	0.71	0.19
HSPG2	0.55	0.2	0.65
DCN	0.33	0.35	0.08
LUM	0.08	0.04	
VCAN	0	0.02	0.02
PRELP	0.02		

Secreted Factors

GeneName	TIF	CAF1	CAF2
S100A13	0.33		
IGF2	0.1	0.06	0.23
S100A6	0.09		0.22
CXCL12	0.09		
S100A10			0.09
SFRP1		0.07	
MDK			0.06
GDF15			0.06
CCL25	0.06		
S100A7		0.05	
S100A9		0.04	
WNT5A		0.04	0.02
FGF5			0.03
FGF2	0.03		
ANGPTL2	0.01		

ECM-affiliated

GeneName	TIF	CAF1	CAF2
LGALS1	9.3	0.08	2.2
ANXA2		0.03	0.81
GREM1	0.09	0.09	0.49
ANXA5	0.08		0.28
ANXA6	0.2		0.02
LGALS3	0.08		
LGALS8	0.03		
CLEC3B		0.02	
C1QTNF3		0.02	

ECM Regulators

GeneName	TIF	CAF1	CAF2
SERPINH1	0.54	0.08	0.02
TGM2	0.08	0.37	0.35
HTRA1	0.12	0.23	0.23
CSTB	0.08		
TIMP3		0.07	
LOXL2	0.07		0.02
CD109	0.06		0.01
CTSB	0.05		0.02
LOX	0.04		
LOXL1	0.03	0.01	
MMP14	0.03		
ADAMTSL4		0.03	
SERPINA1		0.02	
CTSK	0.02		
LOXL4	0.02		
PRSS1		0.02	
ADAMTSL1	0.02	0	
P4HA2	0.01		
PLOD1	0.01		
ITIH2	0.01		0.01

e

	Pearson correlation between LC-MS at the level of Protein Intensity					
	**CAF1.vs.CAF2**	**CAF1.vs.TIF**	**CAF.2.vs.TIF**	**CAF1.vs.CAF1**	**CAF2.vs.CAF2**	**TIF.vs.TIF**
Upper whisker	0.82	0.80	0.88	0.99	0.99	0.98
3rd quartile	0.81	0.78	0.85	0.98	0.98	0.96
**Median**	**0.80**	**0.76**	**0.84**	**0.98**	**0.97**	**0.94**
1st quartile	0.79	0.74	0.83	0.98	0.97	0.92
Lower whisker	0.76	0.71	0.81	0.97	0.96	0.91
Nr. of data points	42.00	42.00	49.00	15.00	20.00	21.00
**Mean**	**0.80**	**0.76**	**0.84**	**0.98**	**0.97**	**0.94**

Characterization of de-cellularised ECM. (a) Scanning electron micrograph of TIF-derived matrix. Scale bar = 1µm **(b)** SDS-PAGE analysis of two independent preparations ECM produced by TIFs plated on non-coated surfaces (-) or, surfaces prepared with a gelatine coating (+) according to the standard protocol. **(c)** Analysis of cell cohort migration on TDM prepared with (+) or without (-) a gelatine surface coating. No significant difference in cell migration distance was observed. **(d)** Different categories of matrisome-associated proteins identified in the de-cellularised TIF and CAF matrices detected by mass spectrometry. The blue histograms and values correspond to the molar % calculated as the ratio of the emPAI score by the total emPAI. **(e)** Comparative analysis of the global proteomes of the different ECM preparations used in this study was performed. Pearson coefficients between the proteins identified by LC-MS are : 0.76 and 0.84 for CAF1 vs TIF and CAF2 vs TIF, respectively.

Supplementary Figure 3

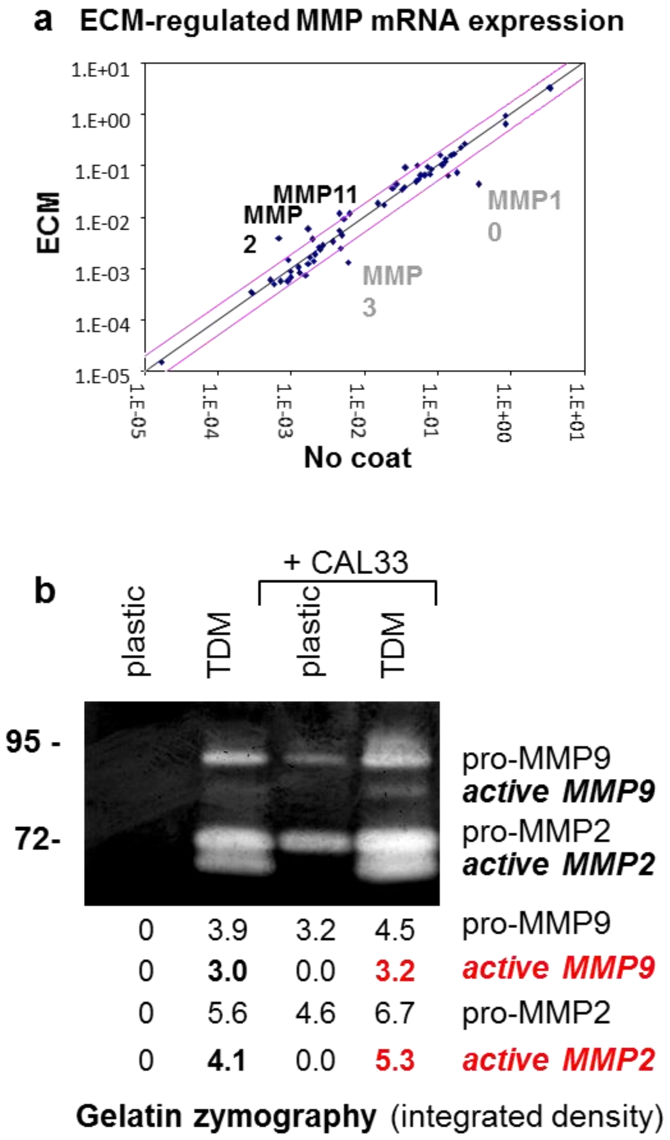

Cell-ECM interactions increase matrix metalloprotease expression and activation. (a) MMP2 and MMP11 mRNA expression is increased in CAL33 cells on TDM. Relative expression comparison for MMP genes between CAL33 cells plated on plastic or TIF-derived ECM (TDM). RNAs were extracted from cells cultured on plastic or TDM using RT2qPCR-Grade RNA isolation kit (SABiosciences, MD, USA). cDNAs were prepared from 500 ng of total RNA with RT2 First Strand Kit (SABiosciences MD, USA) and submitted to real-time PCR analysis using the RT2 profilerTM PCR array system for gene expression profiling of ECM and Adhesion molecules (SABiosciences) using an ABI PRISM® 7900 HT Sequence Detection System (Applied Biosystems, Foster City, CA). Fold-changes in gene expression were calculated for pair-wise comparison using the ΔΔCt method. The analyses were carried out on duplicate samples on two different plates and the experiment was repeated twice. The graph depicts a log transformation plot of the relative expression level of each gene (2-ΔCT) between cells on plastic (x-axis) and cells on TDM (y-axis). The pink lines indicate a 2-fold change in gene expression threshold. **(b)** The effect of adhesion to ECM on secreted MMP activity was determined by gelatin zymography of conditioned medium, performed as described in [1]. A representative gelatin zymography of medium conditioned for 48 hours by CAL33 cells plated on non-coated plastic or TIF-derived ECM is shown (MMP-9: proenzyme = 92kDa/active form = 86kDa; MMP-2 proenzyme = 72kDa/active form = 62kDa). Latent but not active forms of MMP-2 and MMP-9 were secreted by CAL33 cells on plastic. Plating cells on TDM increased MMP-2 expression and activation, and MMP-9 activation. Quantification (below) was performed using ImageJ.

Supplementary Figure 4

a

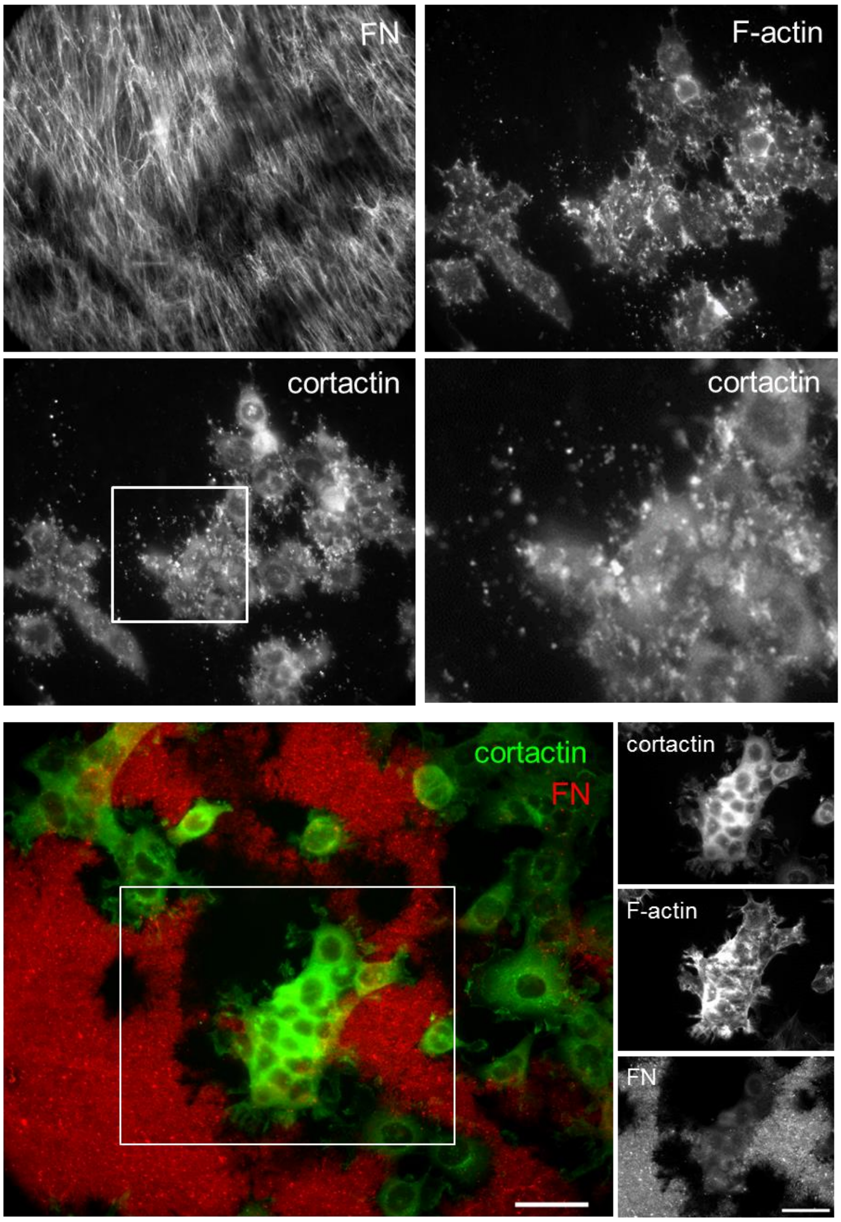

Cell-derived ECM promotes Invadopodial-like structures and tumour cell-derived vesicles.

(a) Representative fluorescence staining of FN, F-actin and cortactin in CAL33 cells plated on TIF-derived matrix (scale bar=20µm). Cortactin (a bona fide component of invadosomes, actin-rich adhesive structures that degrade the ECM via the release of proteases) can be seen in cell-associated structures and in extracellular vesicles sequestered in the fibrillar ECM. Elevated cortactin expression [2] and SFK expression/activity [3] has been observed in HNSCC cell cohorts on ECM. **(b)** Immunofluorescence staining of FN, F-actin and cortactin in CAL33 cells plated for 24 hr on adsorbed plasma FN (10µg/ml). Scale bar = 20µm. The FN substrate beneath cells is efficiently degraded. Cortactin staining is more diffuse in cells on a FN coat than on ECM and it is enriched in peripheral lammelipodia-like structures. Representative images (n≥ 5 fields) from 2 independent experiments are shown.

Supplementary Figure 5

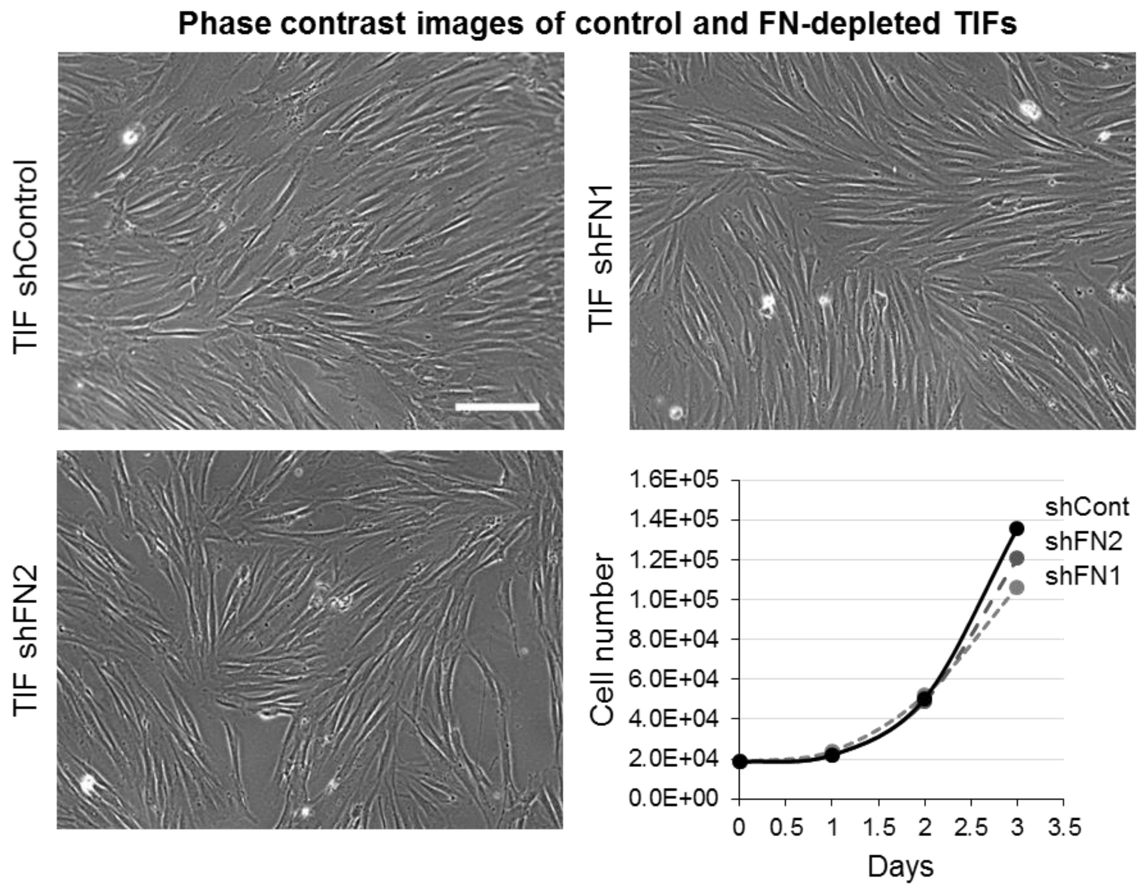

Morphology and proliferation of control and FN-depleted TIFs.
Phase contrast images of the TIFs stably expressing Control (shControl) or FN-targeting shRNA (shFN1 or shFN5). Scale bar = 150µm. Cell proliferation was determined by counting cells 1, 2 or 3 days after seeding them in culture medium supplemented with 20% (v/v) FN-depleted FCS. The mean of triplicate enumerations from a representative experiment (of two) is shown.

Supplementary Figure 6

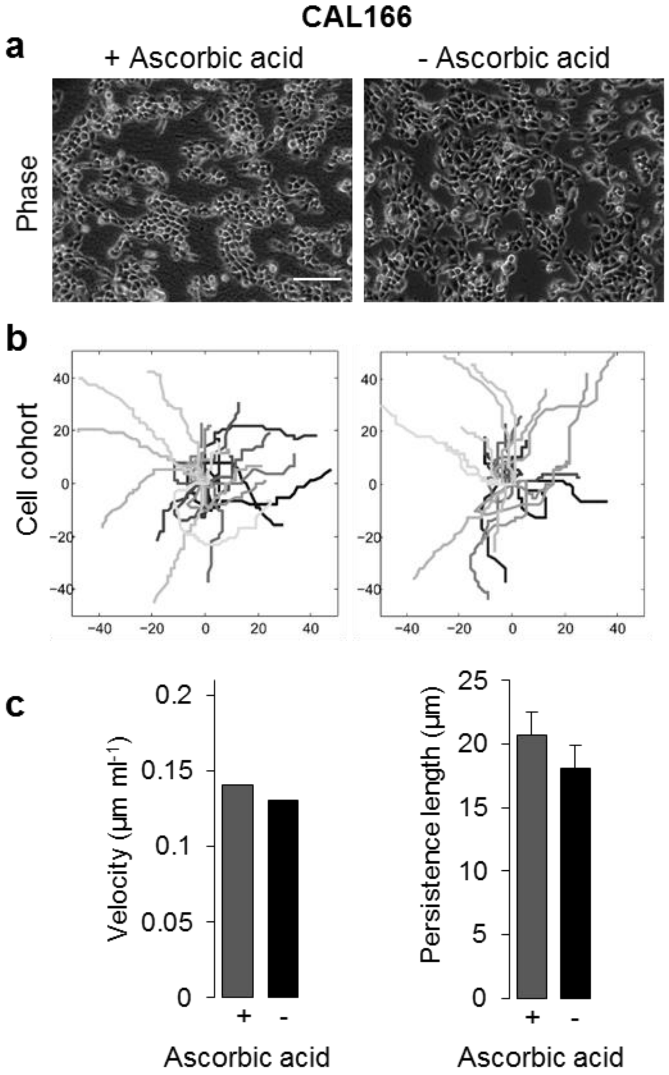

Inhibition of collagen fibrillogenesis does not affect cohort migration of HNSCC cells.

(a) Phase contrast images of the CAL166 HNSCC line plated on TIF-derived ECM produced by cells cultured in presence (+AA) or absence (-AA) of ascorbic acid (Scale bar=150μm). **(b)** Representative tracings (denoted by different grey levels) from origin of cells within clusters seeded on ECM generated by TIF cultured in presence or absence of ascorbic acid. **(c)** Histograms depicting the speed and persistence length of movement from a representative experiment, of at least 2, are shown. Statistical methods are described in Supplementary Experimental Procedures (Analysis of cell migration).

Supplementary Figure 7

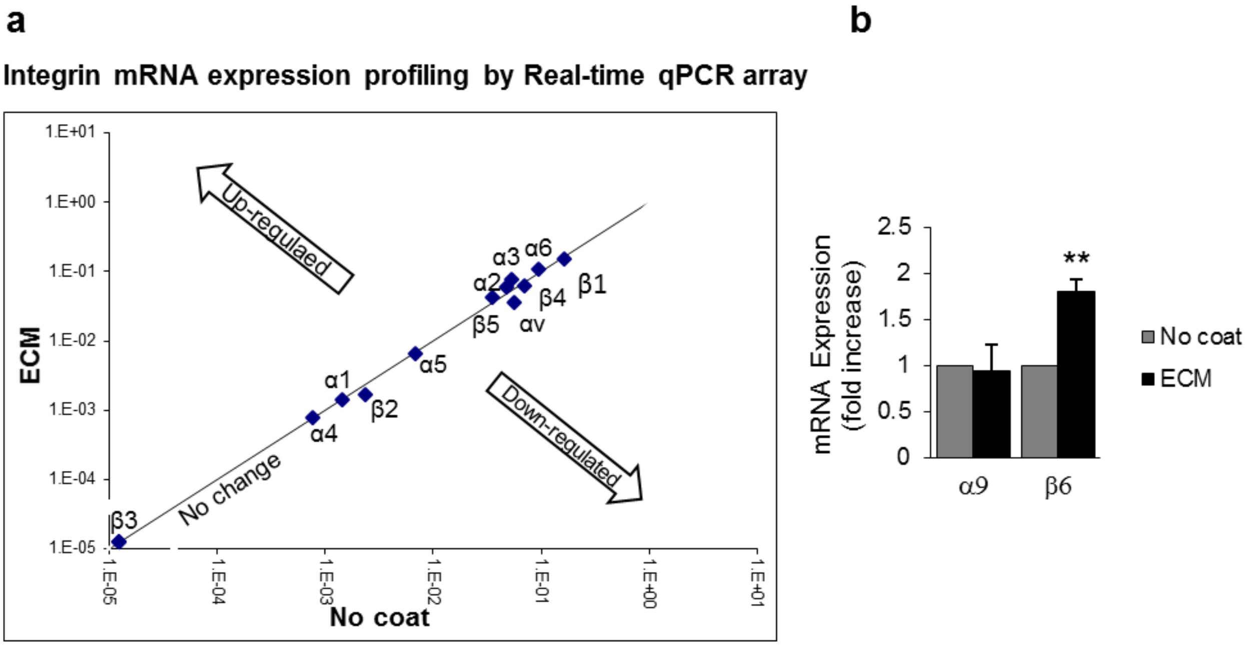

Adhesion to cell-derived ECM has no effect on integrin mRNA expression in HNSCC cells.
(a) The graph depicts a log transformation plot of the relative expression level of each gene (2-ΔCt) between CAL33 cells plated on non-coated plastic (x-axis) or TIF-derived ECM (y-axis). The grey line indicates no change in gene expression threshold. Analyses were carried out using the RT^2 Profiler PCR Array System for gene expression profiling of “ECM and Adhesion Molecules” (SABiosciences) according to the manufacturer’s instructions. Triplicate determinations for each sample were carried out on two separate preparations for each substrate, and the experiment was repeated twice. **(b)** As β6 and α9 integrin subunits were not on present on the “ECM and Adhesion Molecules” RT2 profilerTM PCR array system, we performed separate qPCR analyses to determine the effect of adhesion to TIF-derived ECM on expression of mRNA encoding these integrin subunits in cells. The histogram depicts qPCR analysis (mean +/- s.d. from 3 independent experiments) of α9 and β6 integrin subunit mRNA expression in CAL33 cells seeded for 36 hours on plastic (No coat) dishes or on TIF-derived ECM. Statistically significant data are indicated by *($p<0.05$), ** ($p<0.01$), *** ($p<0.001$) or **** ($p<0.0001$).

Supplementary Figure 8

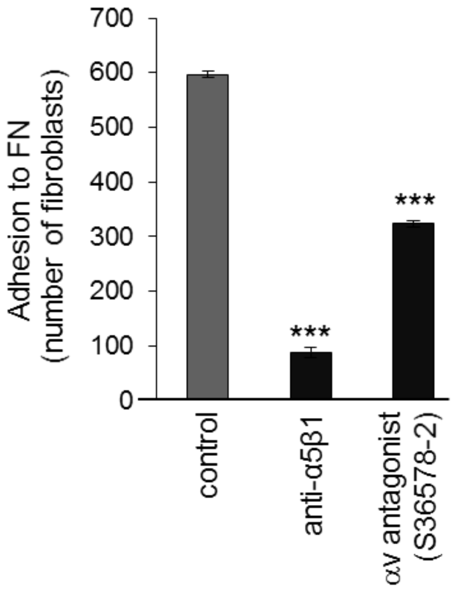

Control of function blocking anti-α5β1 antibodies and the αv integrin antagonist. Inhibition of cell adhesion to FN-coated wells (10µg/ml) was determined 10 min after seeding $5X10^3$ cells in presence of DMSO (control), anti-α5β1 antibody (10µg/ml, clone JBS5) or the S36578-2 αv integrin antagonist (5µg/ml). Histogram depicts adherent cell numbers (mean ±s.d.) from a representative experiment of three. Statistically significant data are indicated by *(p<0.05), ** (p<0.01), *** (p<0.001) or **** (p<0.0001).

Supplementary Figure 9

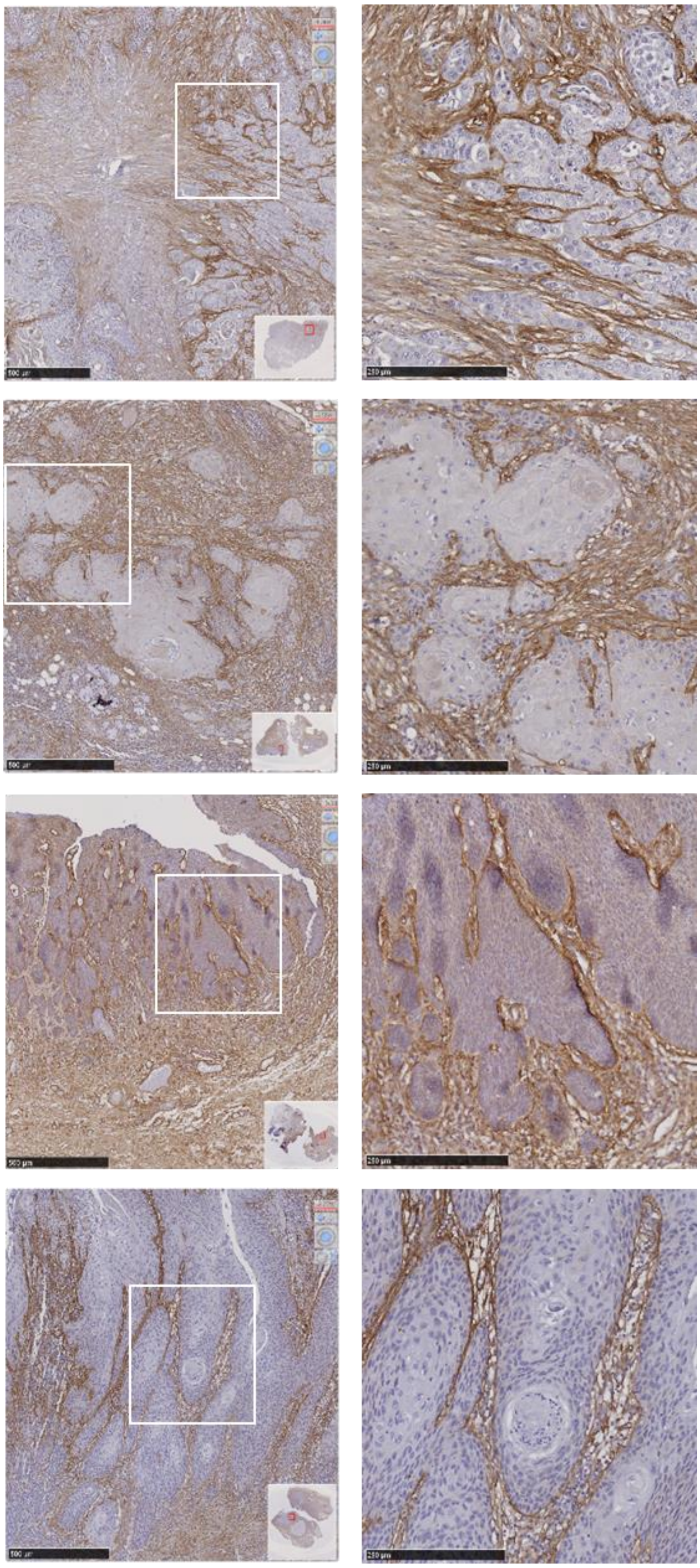

Representative TGFBI staining in whole sections of HNSCC.

Immunohistochemical staining of TGFBI in whole sections from 4 tumours. Images on the right (inserts from left images) correspond to the size of TMA histospots (600µm). Scale bar=250µm for right images and 500µm (top) for left images.

Supplementary Figure 10

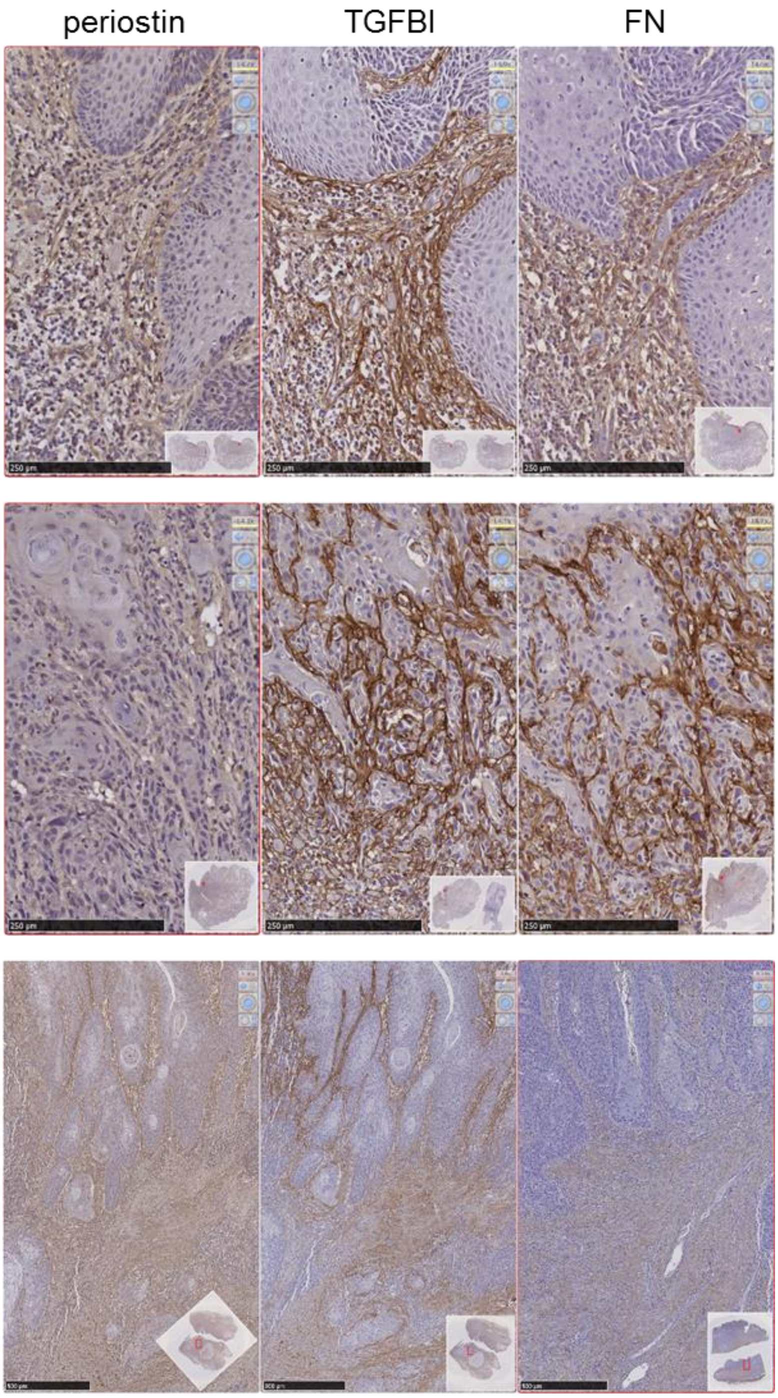

Comparison of periostin, TGFBI and FN staining in HNSCC.
Representative immunohistochemical staining of periostin (left), TGFBI (middle) and FN (right) in whole sections from 3 tumours. The Vectastain ABC signal amplification Kit was used for detection of periostin staining. Scale bar=250µm for top 2 images and 500µm for bottom images.

Supplementary Figure 11

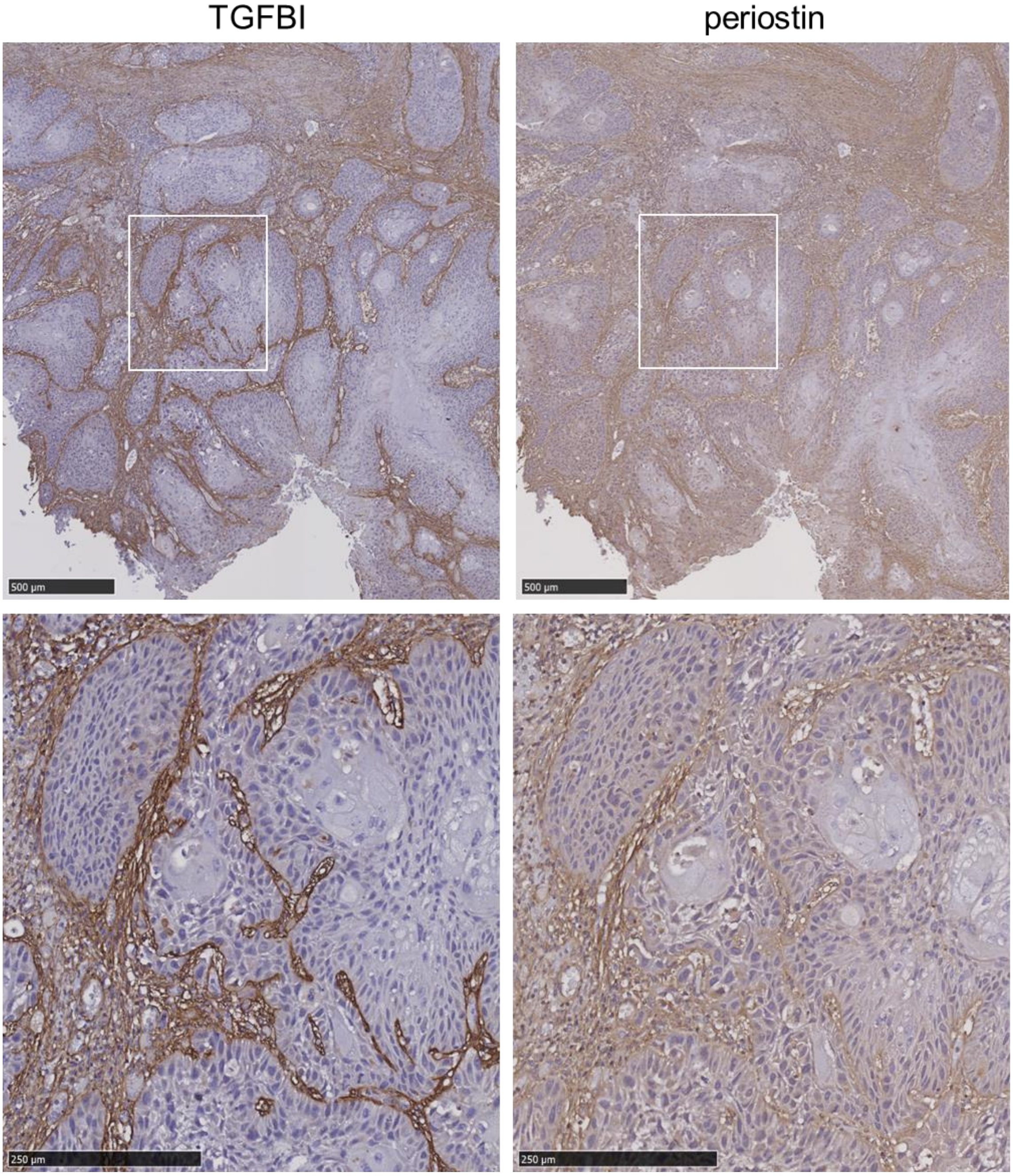

Comparison of periostin and TGFBI staining in HNSCC.

Immunohistochemical staining of periostin (left) and TGFBI (right) in a representative whole tumour section. The Vectastain ABC signal amplification Kit was used for detection of periostin staining. Scale bar=500µm (top) and 500µm for bottom images, which correspond to the size of TMA histospots (600µm).

Supplementary Figure 12

Original immunoblots for indicated figures

Figure 1d

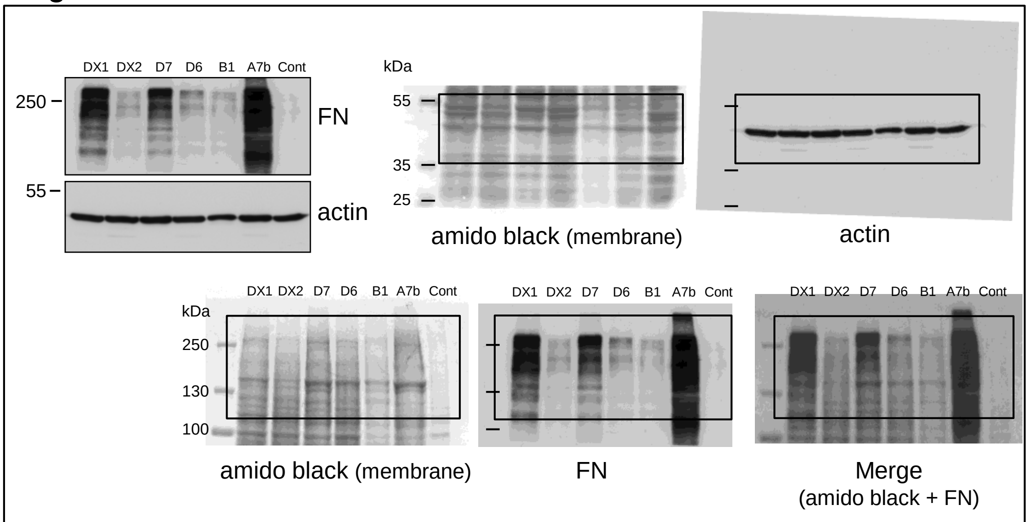

Representative western blots of FN and actin in human tumor lysates

Figure 1e

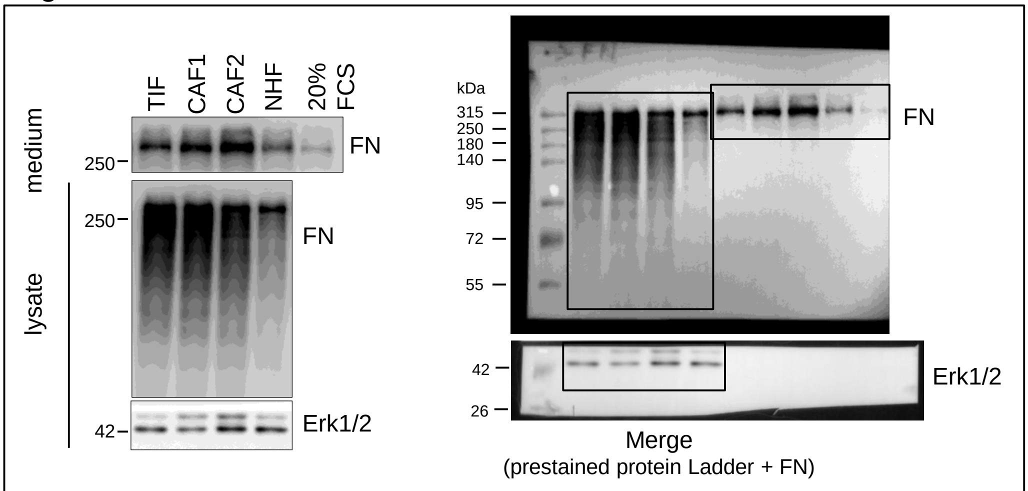

Representative western blots of FN and ERK1/2 in fibroblast lysates and conditioned medium

Supplementary Figure 13

Original immunoblots for indicated figures

Figure 6

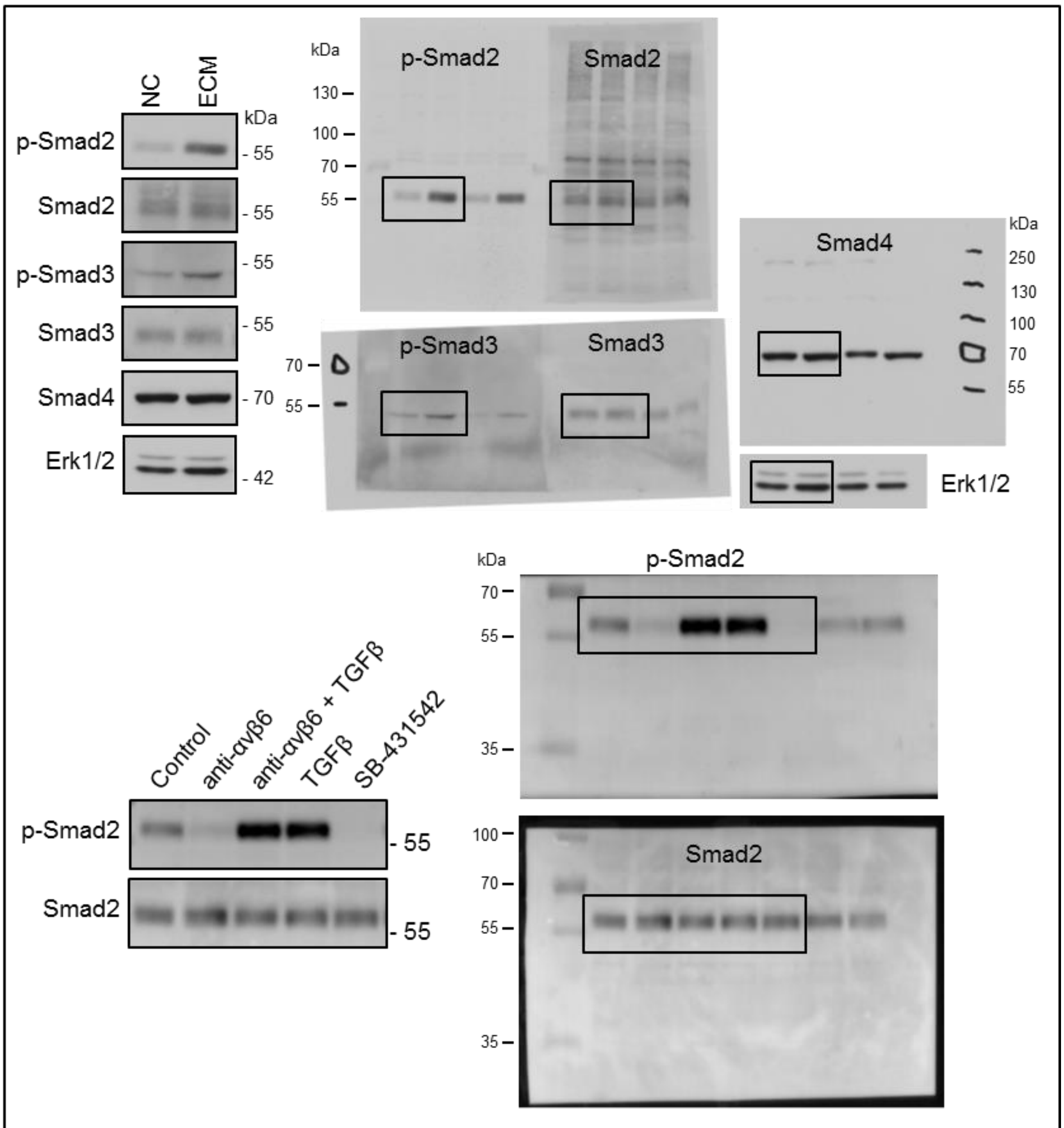

Representative western blots of indicated Smads/phospho-Smads

Supplementary Figure 14

Original immunoblots for indicated figure

Figure 6

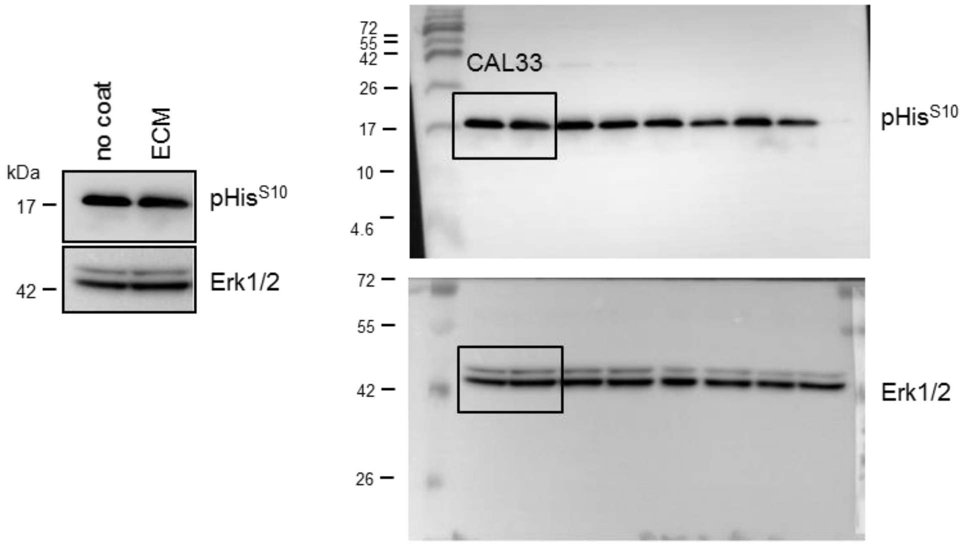

Representative western blots of p-His (Ser10) and ERK1/2 in HNSCC lines

Figure 7

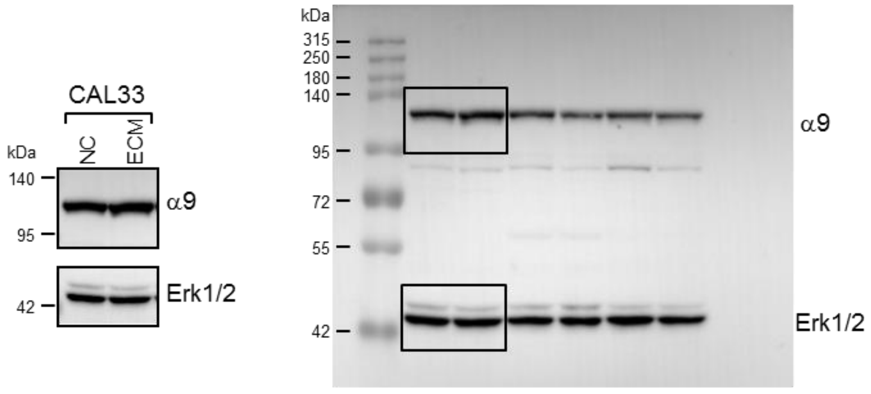

Representative western analysis of α9 integrin and ERK1/2 expression in HNSCC lines

Supplementary Table 1

Primary antibodies used, suppliers and dilutions

Antibody	Clone(Reference)	Supplier (address)	Source	Dilution
Smad2	(5339)	Cell Signalling Technology (Beverly, MA)	rabbit	1:1000
phospho-Smad2 (Ser465/467)	(3108)	Cell Signalling Technology	rabbit	1:1000
Smad3	(9523)	Cell Signalling Technology	rabbit	1:1000
phospho-Smad3 (Ser423/425)	(9520)	Cell Signalling Technology	rabbit	1:1000
FN	(610077)	BD Biosciences (Le Pont de Claix, France)	mouse	WB 1:4000<br>IHC 1:2000
E-cadherin	(610181)	BD Biosciences	mouse	1:100
TNC	BC24 (T2551)	Sigma-Aldrich (St. Louis MO)	mouse	1:2000
αSMA	1A4 (A2547)	Sigma-Aldrich	mouse	1:500
β actin	AC-15 (A5441)	Sigma-Aldrich	mouse	1:200
periostin	(HPA012306)	Sigma-Aldrich	rabbit	1:100
collagen I	(ab6308)	Abcam (Cambridge, MA)	mouse	1:1000
α9β1 integrin	Y9A2 (MAB2078)	Abcam	mouse	blocking 10μg ml^{-1}
phospho-Histone H3 (Ser10)	(ab5176)	Abcam	rabbit	1:5000
TGFBI	(ab169771)	Abcam	rabbit	1:250
FN-EDA	IST-9 (S-FN5)	Sirius biotech (Genoa, Italy)	mouse	IHC 1:25<br>IF 1:100
ERK1	C-16 (SC-93)	Santa Cruz Biotechnology (Santa Cruz, CA)	rabbit	1:2000
FN	(ab1945)	Millipore (Billerica, MA)	rabbit	IF 1:400
αvβ3 integrin	LM609 (MAB1976)	Millipore	mouse	FACS 1:30
α5β1 integrin	(MAB1999)	Millipore	mouse	FACS 1:75
α5β1 integrin	JBS5 (MAB1965)	Millipore	mouse	blocking 10μg ml^{-1}
αvβ5 integrin	P1F6 (MAB1961)	Millipore	mouse	blocking 20μg ml^{-1}<br>FACS 1:50
αvβ6 integrin	E7P6	Millipore	mouse	FACS 1:75
collagen VI	(MAB3303)	Millipore	mouse	1:200
cortactin p80/85	4F11 (05-180)	Millipore	mouse	1:500
α9β1 integrin	(MAB2078)	Millipore	mouse	blocking 10μg ml^{-1}<br>FACS 1:50
β1 integrin	lia1/2 (20-511)	GenWay Biotech (San Diego, CA)	mouse	FACS 1:10
β1 integrin	P5D2 (MAB17781)	R&D systems (Abingdon, UK)	mouse	blocking 10μg ml^{-1}
α9 integrin	(PA5-27771)	Thermo Scientific (Rockford, IL)	rabbit	WB 1:1000<br>IHC 1:100
CD31	JC70A	Dako	mouse	IHC 1:1000
αvβ6 integrin	6.3G9	produced in-house	mouse	blocking 45μg ml-1
αvβ6 integrin	6.2A1	produced in-house	mouse	IHC 5μg ml-1

Supplementary References

1. Hamaguchi M*, et al.* Augmentation of metalloproteinase (gelatinase) activity secreted from Rous sarcoma virus-infected cells correlates with transforming activity of src. *Oncogene* **10**, 1037-1043 (1995).

2. Hofman P*, et al.* Prognostic significance of cortactin levels in head and neck squamous cell carcinoma: comparison with epidermal growth factor receptor status. *Br J Cancer* **98**, 956-964 (2008).

3. Veracini L*, et al.* Elevated Src family kinase activity stabilizes E-cadherin-based junctions and collective movement of head and neck squamous cell carcinomas. *Oncotarget* **6**, 7570-7583 (2015).
